# Supplementary material for: A panoptic segmentation dataset and deep-learning approach for explainable scoring of tumor-infiltrating lymphocytes
Source: NPJ Breast Cancer. 2024 Jun 28;10:52. doi: 10.1038/s41523-024-00663-1 (PMC11213912; doi:10.1038/s41523-024-00663-1)
Supplement: Supplementary file 1 — Supplement [file 41523_2024_663_MOESM1_ESM.pdf]

Supplementary Material

A panoptic segmentation dataset and deep-learning approach for explainable scoring of tumor-infiltrating lymphocytes

Shangke Liu[1†], Mohamed Amgad[1†*], Deeptej More[1], Muhammad A. Rathore[1], Roberto Salgado[2,3], Lee A.D. Cooper[1*]

[1] Department of Pathology, Northwestern University, Chicago, IL, USA [2] Department of Pathology, GZA-ZNA Ziekenhuizen, Antwerp, Belgium, [3] Division of Research, Peter MacCallum Cancer Centre, Melbourne, Australia.

† Co-first authors.
*** Address correspondence to: *lee.cooper@northwestern.edu* and *mohamed.tageldin@nm.org.***

Supplementary Table 1. **Total number of nuclear annotations in PanopTILs dataset.**

Nuclei annotation type	Count
**Cancer**	16322
**Lymphocyte**	9596
**Fibroblast**	6945
**Debris**	5943
**Plasma Cell**	4641
**Active Stromal Cell**	1041
**Normal epithelium**	382
**Other Cell**	3

Supplementary Table 2. **Impact of region constraint on accuracy of nuclei classifications.** Bolded values indicate higher performance. Utilizing region constraints improves accuracy for most categories in most test folds. Notably, region constraints improve classification accuracy for the Epithelium and Fibroblast classes.

	Fold1	Fold2	Fold3	Fold4	Fold5
**Accuracy with region constraint**					
**Epithelial**	**87.3**	**84.2**	**92.5**	**88.1**	**88.4**
**Stromal**	**84.7**	**81.1**	**87.2**	**82.4**	**84.0**
**TIL**	**90.7**	**89.9**	**92.0**	**88.9**	**90.8**
**Debris**	**95.5**	93.9	**97.1**	**95.5**	97.0
**Accuracy without region constraint**					
**Epithelial**	83.2	83.6	91.6	83.2	83.7
**Stromal**	82.7	81.0	87.1	79.0	82.5
**TIL**	89.5	88.6	91.8	86.9	90.0
**Debris**	95.4	**95.8**	96.8	95.2	**97.4**

Supplementary Table 3. **Impact of region constraint on Matthews Correlation Coefficient of nuclei classifications.** Bolded values indicate higher performance. Utilizing region constraints improves MCC for all categories in all test folds. Using region constraints improves MCC for Epithelial and Fibroblast classes. Performance improvements for debris are considerable.

	Fold1	Fold2	Fold3	Fold4	Fold5
**MCC with region constraint**					
**Epithelial**	**74.4**	**69.5**	**84.6**	**73.5**	**76.3**
**Stromal**	**60.5**	**54.4**	**67.4**	**57.7**	**61.0**
**TIL**	**74.9**	**73.7**	**80.0**	**75.7**	**77.0**
**Debris**	**36.1**	**35.5**	**57.2**	**44.4**	**27.7**
**MCC without region constraint**					
**Epithelial**	66.9	67.2	82.9	64.2	67.9
**Stromal**	53.4	53.7	67.0	48.3	55.2
**TIL**	72.3	71.4	79.7	72.0	75.0
**Debris**	17.5	0.0	44.6	1.7	-0.2

Supplementary Table 4. **Impact of region constraint on AUROC of nuclei classifications.** Bolded values indicate higher performance. Utilizing region constraints improves MCC for all categories in all test folds.

	Fold1	Fold2	Fold3	Fold4	Fold5
**AUROC with region constraint**					
**Epithelial**	**94.0**	**93.7**	**97.3**	**94.0**	**95.5**
**Stromal**	**90.0**	**87.7**	**93.2**	**88.3**	**90.4**
**TIL**	**96.2**	**96.4**	**97.6**	**96.2**	**96.8**
**Debris**	**84.6**	**86.2**	**93.4**	**89.9**	**87.7**
**AUROC without region constraint**					
**Epithelial**	91.0	91.6	97.0	90.4	92.6
**Stromal**	87.9	85.9	92.9	84.4	88.4
**TIL**	95.3	95.5	97.5	94.8	96.0
**Debris**	80.0	75.9	93.3	73.8	77.4

Supplementary Table 5. **Impact of region constraint on precision and recall of nuclei classifications.** Results are shown in precision/recall pairs. Bolded values indicate higher performance (average of precision and recall). In many instances, using region constraints improves precision markedly with only a small tradeoff in recall.

	Fold1	Fold2	Fold3	Fold4	Fold5
**Precision / recall with region constraint**					
**Epithelial**	**84.4 / 87.7**	**92.9 / 71.6**	**90.8 / 91.5**	**84.4 / 80.5**	**84.1 / 88.2**
**Stromal**	**71.3 / 70.5**	**61.8 / 73.3**	**71.2 / 81.0**	**74.0 / 66.3**	**69.6 / 75.1**
**TIL**	**80.1 / 82.1**	**72.8 / 88.2**	**89.9 / 81.2**	**75.3 / 92.2**	**87.0 / 79.5**
**Debris**	**49.8 / 29.4**	**32.9 / 45.3**	**62.5 / 55.3**	**53.8 / 40.6**	**38.2 / 22.1**
**Precision / recall without region constraint**					
**Epithelial**	77.4 / 88.0	87.5 / 75.4	87.9 / 92.8	73.2 / 81.6	75.4 / 88.7
**Stromal**	70.7 / 59.0	61.7 / 72.1	71.4 / 80.0	70.9 / 54.5	70.2 / 64.2
**TIL**	76.0 / 82.7	69.0 / 89.3	90.2 / 80.4	71.3 / 91.9	84.2 / 79.6
**Debris**	44.8 / 8.0	0.0 / 0.0	64.1 / 33.0	29.2 / 0.1	0.0 / 0.0

Supplementary Table 6. **Impact of region constraint on F1 score of nuclei classifications.** Bolded values indicate higher performance. Utilizing region constraints improves F1 score for all categories in all test folds except for 1 (Fold 2, Epithelial), where the difference is only 0.01.

	Fold1	Fold2	Fold3	Fold4	Fold5
**F1 score with region constraint**					
**Epithelial**	**86.0**	80.9	**91.1**	**82.4**	**86.1**
**Stromal**	**70.9**	**67.1**	**75.8**	**69.9**	**72.2**
**TIL**	**81.1**	**79.8**	**85.3**	**82.9**	**83.1**
**Debris**	**37.0**	**38.1**	**58.6**	**46.3**	**28.0**
**F1 score without region constraint**					
**Epithelial**	82.4	**81.0**	90.3	77.2	81.5
**Stromal**	64.3	66.5	75.4	61.6	67.0
**TIL**	79.2	77.8	85.0	80.3	81.9
**Debris**	13.6	0.0	43.6	0.3	0.0

Supplementary Table 7. **Impact of region constraint on specificity and sensitivity of nuclei classifications.** Bolded values indicate higher performance (average of sensitivity and specificity). Utilizing region constraints often improves sensitivity with a modest tradeoff in specificity for most classes and in most folds.

	Fold1	Fold2	Fold3	Fold4	Fold5
**Specificity / sensitivity with region constraint**					
**Epithelial**	**86.9 / 87.7**	**95.2 / 71.6**	**93.2 / 91.5**	**92.1 / 80.5**	**88.6 / 88.2**
**Stromal**	**89.8 / 70.5**	**83.9 / 73.3**	**89.3 / 81.0**	**89.6 / 66.3**	**87.4 / 75.1**
**TIL**	**93.5 / 82.1**	**90.4 / 88.2**	**96.3 / 81.2**	**87.6 / 92.2**	**95.3 / 79.5**
**Debris**	**98.6 / 29.4**	**96.0 / 45.3**	**98.7 / 55.3**	**98.2 / 40.6**	**99.0 / 22.1**
**Specificity / sensitivity without region constraint**					
**Epithelial**	79.3 / 88.0	90.6 / 75.4	90.6 / 92.8	84.1 / 81.6	80.3 / 88.7
**Stromal**	91.2 / 59.0	84.1 / 72.1	89.5 / 80.0	90.0 / 54.5	89.5 / 64.2
**TIL**	91.7 / 82.7	88.4 / 89.3	96.5 / 80.4	84.8 / 91.9	94.1 / 79.6
**Debris**	99.5 / 8.0	100 / 0.0	99.3 / 33.0	100 / 0.1	100 / 0.0

Supplementary Table 8. **Performance comparison of tissue region segmentation of MuTILs versus the VGG-FCN8 model described in [12]**. Bolded values indicate higher performance. For a fair comparison only slides present in the testing set(s) of both models were used. Note that VGG-FCN8 model segments slides at a 40x magnification, while MuTILs is trained to segment slides at a 10x magnification to provide low-power context for the nucleus classifications. While this results in some drop in accuracy of the cancer and TILs-dense region segmentation, the lower power context improves stromal region classification.

	Epithelial	Stromal	TILs
**MuTils tissue segmentation (10X magnification)**			
**DICE overall**	86.8	**85.9**	70.2
**DICE slide average (std)**	82.1 (13.2)	**82.3 (8.9)**	62.4 (21.4)
**VGG-FCN8 tissue segmentation (40X magnification)**			
**DICE overall**	**89.1**	82.2	**77.3**
**DICE slide average (std)**	**86.8 (7.9)**	77.9 (12.2)	**70.0 (22.6)**

Supplementary Table 9. Performance comparison of nuclei classification of MuTILs versus the mask-RCNN model described in [13]. Bolded values indicate higher performance. Assignment to training/testing folds was the same in both works, allowing exact comparison. Mean and standard deviation statistics exclude fold 1, which contributed to model turning. MuTILs outperforms the mask-RCNN model for all nuclei types evaluated.

	**Fold1**	**Fold2**	**Fold3**	**Fold4**	**Fold5**	**Mean (std)**
			**MuTILs MCC**			
**Epithelial**	**74.4**	69.5	**84.6**	73.5	**76.3**	**76.0 (6.4)**
**Stromal**	**60.5**	**54.4**	**67.4**	**57.7**	**61.0**	**60.1 (5.5)**
**TIL**	**74.9**	73.7	**80.0**	75.7	**77.0**	**76.6 (2.6)**
			**mask-RCNN MCC**			
**Epithelial**	72.9	**73.7**	74.9	**80.6**	57.4	71.7 (10.0)
**Stromal**	47.1	53.0	46.9	56.9	40.7	49.4 (7.1)
**TIL**	73.7	**76.6**	77.9	**79.6**	60.1	73.5 (9.1)
			**MuTILs AUROC**			
**Epithelial**	94.0	93.7	**97.3**	94.0	**95.5**	**95.1 (1.7)**
**Stromal**	**90.0**	**87.7**	**93.2**	88.3	**90.4**	**89.9 (2.5)**
**TIL**	**96.2**	**96.4**	**97.6**	**96.2**	**96.8**	**96.8 (0.6)**
			**mask-RCNN AUROC**			
**Epithelial**	**94.2**	**94.5**	96.1	**97.2**	88.8	94.2 (3.7)
**Stromal**	83.2	87.4	84.3	**89.1**	80.7	85.4 (3.7)
**TIL**	95.3	96.2	95.7	95.9	91.0	94.7 (2.4)

Supplementary Figure 1. Distribution of No of TILs / Total No of cells. The distribution of the "No of TILs / Total No of cells" (nTnA) TIL Score. This score includes all cells in the denominator, and so the 10% threshold used for stromal scores is too conservative. The three leftmost histogram bins encompass around 50% of the total patients. Based on our observation, we selected a threshold value of 3% as it roughly represents the midpoint of this cumulative distribution where half of the patients lie. A 10% threshold would result in a significant imbalance and make comparison between the different scores difficult. The accompanying summary table further emphasizes the distribution of samples, highlighting that out of 304 total samples, 161 samples are above the 3% mark and 143 samples are below it.

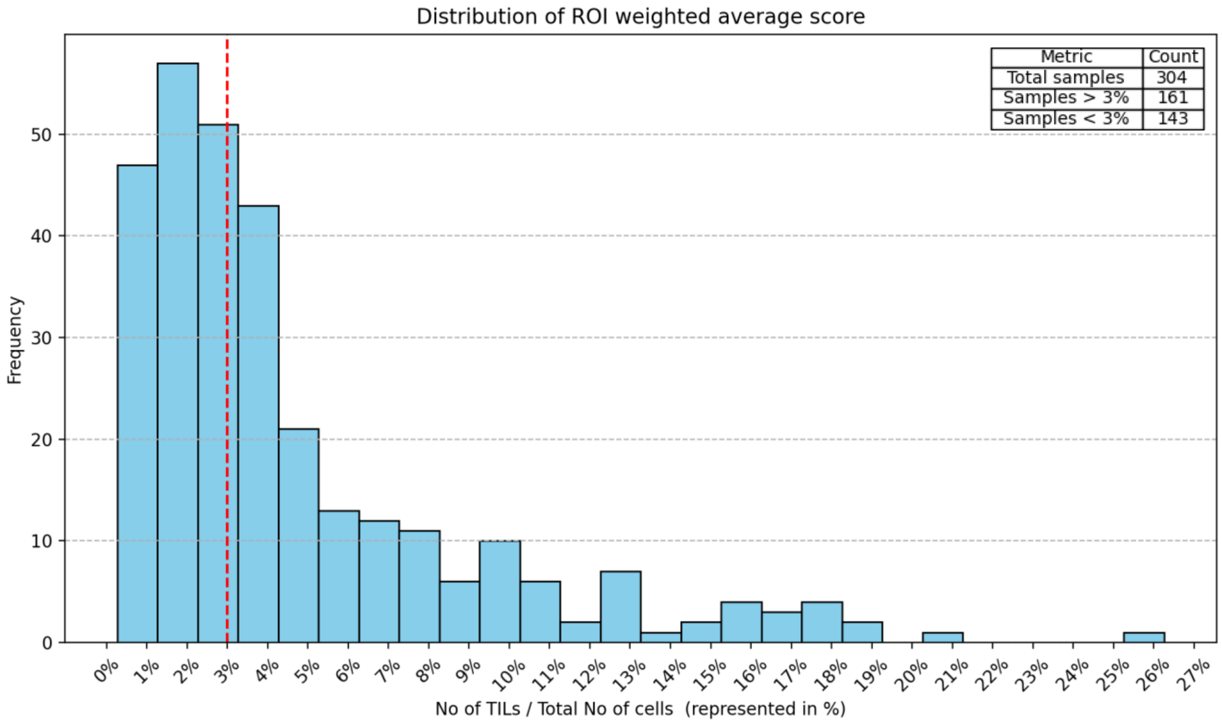

Supplementary Figure 2. Confusion matrix of MuTIL nuclear classification. Values represent predictions aggregated over all samples and validation folds. Plots in the top row present classification counts where plots in the bottom row present percentages calculated for each ground truth label. **a.** The region constraint improves classification of stromal (fibroblast) and debris nuclei. **b.** Without region constraint, classification of epithelial nuclei improves at the cost of misclassifications for stromal and debris nuclei.

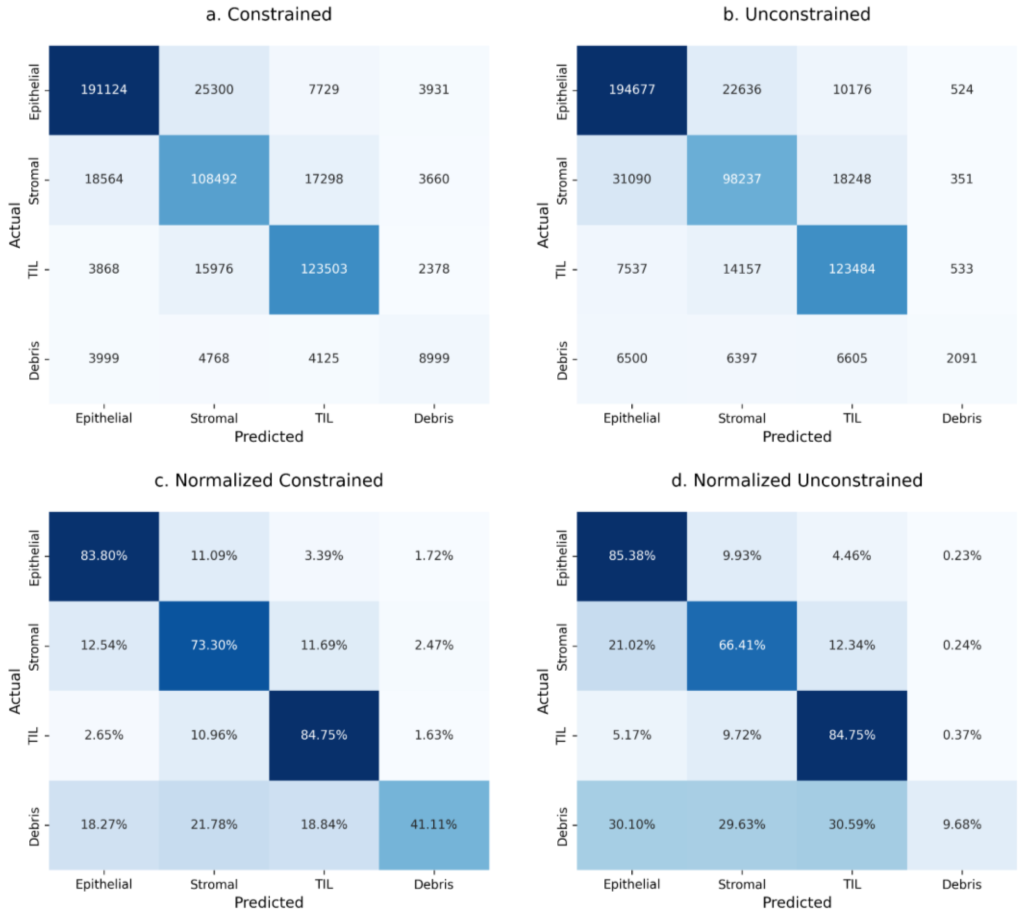

Supplementary Figure 3. Bland-Altman plots between pathologists. Most points lie within +/- two standard deviation interval. Outliers are in the moderate score range from %20-%60. Most outliers indicate a higher bias for pathologist 2. No proportional bias is observed in higher scoring cases.

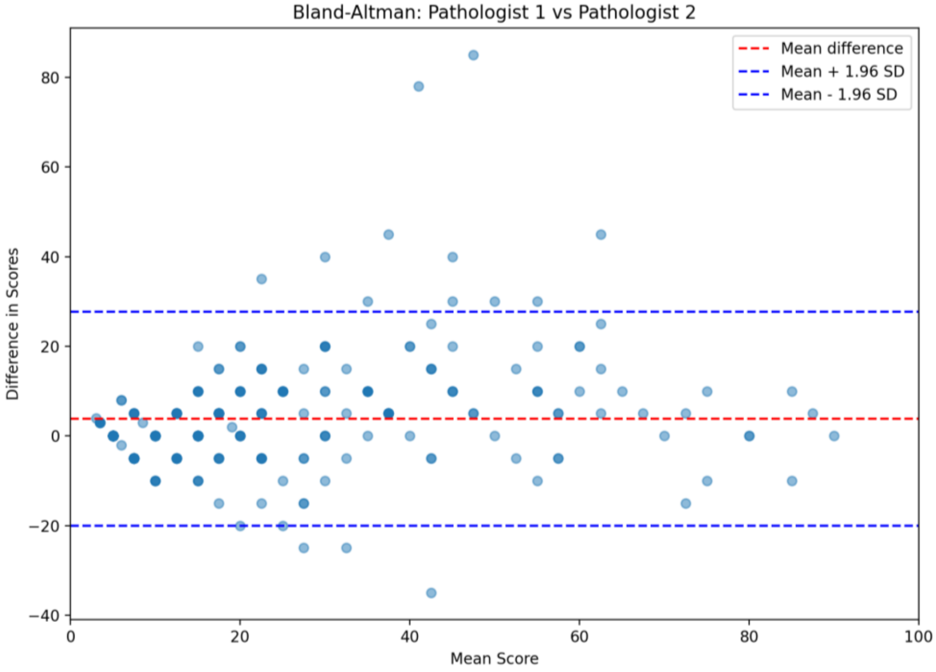

Supplementary Figure 4. Heatmap computational TIL score variant correlation. Spearman correlations were calculated for each combination of score variants (nTSa, nTnS, nTnA) and score aggregation method (global, saliency weighted). For each variant, correlations between the global and saliency weighted scores are high, ranging from 0.89-0.92. Across variants, correlations are lower but still high, ranging from 0.72 to 0.86. It's noteworthy that within each scoring category—whether focusing on stromal area, number of cells in stroma, or total number of cells—the global and ROI average scores consistently show high correlation. This highlights the reliability and coherence of the TIL score measurements.

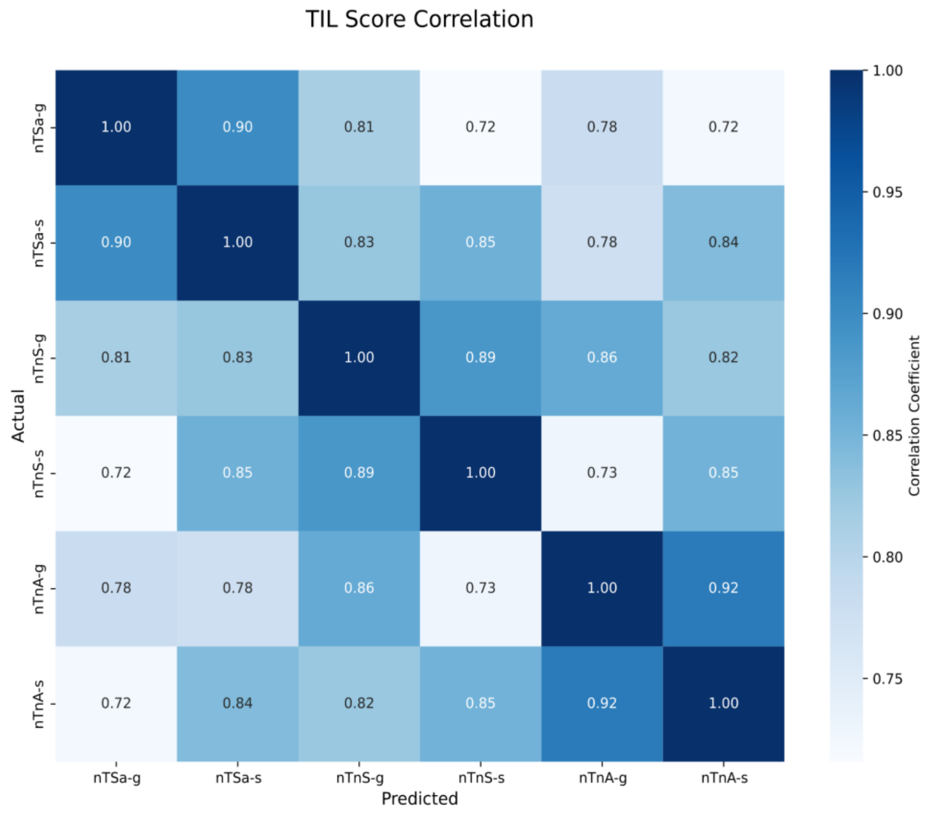

Legend:

Key	Computational score	Aggregation
**nTSa-g**	nTSa	Global
**nTSa-s**	nTSa	Saliency-weighted
**nTnS-g**	nTnS	Global
**nTnS-s**	nTnS	Saliency-weighted
**nTnA-g**	nTnA	Global
**nTnA-s**	nTnA	Saliency-weighted
